# Cloning and characterization of the N-methyl-D-aspartate receptor subunit NR1 gene from chum salmon, *Oncorhynchus keta* (Walbaum, 1792)

**DOI:** 10.1186/2193-1801-3-9

**Published:** 2014-01-03

**Authors:** Jeong-Nam Yu, Seung Hyub Ham, Seung Il Lee, Hyung-Joo Jin, Hiroshi Ueda, Deuk-Hee Jin

**Affiliations:** Department of Marine Molecular Biotechnology, Gangneung-Wonju National University, Gangneung, 210-702 Korea; Laboratory of Aquatic Bioresources and Ecosystem, Field Science Center for Northern Biosphere, Hokkaido University, North 9, West 9, Kita-ku, Sapporo, 060-0809 Japan; National Institute of Biological Resources, Environmental Research Complex, Incheon, 404-708 Korea

**Keywords:** Olfactory, N-methyl-D-aspartate receptors, *Oncorhynchus*, NR1 gene

## Abstract

Here, we report the information about molecular and expression characterization of NR1 gene in chum salmon for the first time. The complete NR1 subunit showed a large open-reading frame of 2844 bp in the total length of 3193 bp, and this cDNA contained a coding region encoding 948 amino acids and a stop codon. The organization of the NR1 subunit of chum salmon were similar of most other fishes, except C’ terminal. The expression of NR1 subunit was to show higher in the natal river near to the hatchery than near to the coast. We expect that the information reported herein may facilitate further investigations on the relationship between memory factors of natal rivers and homing mechanisms in Salmonidae.

## Introduction

Salmon are an important biological and economic resource in countries of the North Pacific Rim and Asia (Groot and Margolis 1991). Pacific salmon are anadromous fish of the genus *Oncorhynchus* that spend most of their lives rearing in the ocean before returning to freshwater to spawn (Groot and Margolis 1991). There are seven species of Pacific salmon: sockeye (*O. nerka*), pink (*O. gorbuscha*), chum (*O. keta*), chinook (*O. tshawytscha*), coho (*O. kisutch*), masu (*O. masou*), and amago/biwamasu (*O. thodurus*). Among Pacific salmon in Korea, most chum and masu return to their natal river (Groot and Margolis 1991).

Pacific salmon have an amazing ability to migrate thousands of kilometers from the open ocean to their natal stream for reproduction after several years of oceanic feeding (Quinn and Groot 1984). It is now widely accepted that specific factors of the natal stream are imprinted into the nervous systems of juvenile salmon during downstream migration and that adult salmon evoke these factors to recognize the natal stream during spawning and upstream migrations (Ueda 2011). In anadromous salmonids, olfaction is essential for successful completion of homing migration (Hasler and Scholz 1983). Recent electrophysiological studies have clearly shown that amino acids play an important role in the homing behavior of salmonid fish (Shoji et al. 2000). According to Putman et al. ( 2013), to test the hypothesis that salmon imprint on the magnetic field that exists where they firstly enter the sea and later seek the same field upon return using 56 year fisheries date. In the results, they were suggests that salmon homing may be accomplished by imprinting oncharacteristics of the Earth’s magnetic field. However, how the olfactory system discriminates among various stream odors and which sensory systems play leading roles in open water orientation are still unclear.

Studies on the formation of memory have recently concentrated on the possible role of N-methyl-D-aspartate receptors (NMDARs) (Cull-Candy et al. 2001; Dingledine et al. 1999; Hua and Smith 2004; Waxman and Lynch 2005). The NMDARs are ligand-gated iontropic glutamate receptors that are important mediators for neuronal events such as synaptic plasticity, learning and memory, neuronal development, and circuit formation. They have been implicated in various neuronal disorders (Cull-Candy et al. 2001; Dingledine et al. 1999; Hua and Smith 2004; Waxman and Lynch 2005). NMDARs are heteromers comprising two obligate NMDARs and two NR2 (NR2A-D) or NR3 subunits (NR3A-B) (Cull-Candy and Leszkiewicz 2004). Teleost NMDARs have been cloned from knifefish (*Apteronotus leptorpynchus*), rainbow trout (*O. mykiss*), and zebrafish (*Danio rerio*) (Bottai et al. 1997; Harvey-Girard and Dunn 2003; Cox et al. 2005; Kinoshita et al. 2005). However, in anadromous salmon, only sockeye salmon was registered partial NMDA receptor genes sequence in the NCBI database, but the authors has not published English papers.

In the present study, we analyzed the complete sequence and structure of the NR1 subunit in the chum salmon and evaluated its relationship to that of other fish species. We propose evidence of the presence of the NR1 subunit in the central nervous system of chum salmon for the first time in Korea.

## Material and methods

### Fish samples

Individual of chum salmon (*O. keta*) were collected from Yangyang of South Korea from the home river site for analyzing of the complete sequence and structure in the NR1 subunit. Also, we were collected average 10 individuals per each site from Korea for testing of expression of NR1 gene from 2012. All brain tissue was store at -80°C prior to analysis.

### Total RNA isolation and cDNA synthesis

Total RNA was isolated from brain tissue using a Trizol reagent (Invitrogen, Carlsbad, CA, USA) and following the manufacturer’s instructions, including performance of a DNase treatment. Briefly, a maximum of 50–100 μg tissue with Trizol reagent was used per total RNA extraction. The tissues were homogenized using a tissue homogenizer, followed by centrifugation at 12,000 rpm for 15 min at 4°C. Total RNA was precipitated from the aqueous phase by adding an equal volume of isopropanol and centrifuging at 12,000 rpm for 10 min at 4°C. The total RNA pellet was washed twice with 250 μL diethyl pyrocarbonate-treated 75% ethanol. All total RNA samples were subjected to DNase digestion to remove any residual genomic DNA contamination. Finally, the total RNA was dissolved in 20 μL diethyl pyrocarbonate-treated H_2_O and stored at -80°C after checking the quality of total RNA on a 1.5% agarose gel and via UV spectrophotometry (Bio-Rad Laboratories, Hercules, CA, USA).

The first-strand cDNA was synthesized using 0.5 μL DNase-treated total RNA using a SuperScript II Kit (Invitrogen, Carlsbad, CA, USA) with the β-actin primer. The total volume of the reverse transcriptional system was 4 μL (0.5 μL dNTP mix [10 mM of each]), 2 μL 5× reaction buffer, 0.5 μL RNase inhibitor [20 U/μL], and 0.5 μL reverse transcriptase [200 U/μL]). The procedure was carried out according to the manufacturer’s protocol. The cDNA was stored at -20°C, and we confirmed the pre-PCR of cDNA using the *β-actin* gene of trout.

### Reverse transcription (RT)-PCR amplification

Three sets of primers for amplification of the NR1 subunit in chum salmon were designed using the NR1 subunit from the NCBI database (*Oreochromis niloticus*, *Apteronotus leptorhynchus*, *Carassius carassius*, *Danio rerio*, and *O. mykiss*) (Table 1). The primer information is shown in Table 1. The conditions of RT-PCR amplification using the AccuPower® PCR PreMix Kit (Bioneer Co., Seoul, Korea) and the GeneAmp PCR System 2400 (Applied Biosystems, Foster City, CA, USA) were as follows: initial denaturation at 94°C for 10 min, followed by 35 cycles of denaturation 94°C for 1 min, annealing at 55°C for 1 min, and extension at 72°C for 1 min, with a final extension at 72°C for 10 min. The quality was checked on a 2% agarose gel and by spectrophotometry (Bio-Rad Laboratories, Hercules, CA, USA).

**Table 1:** **Number of primer sets and information of primer sets for PCR and sequencing**

Number of primer sets	Primer name	Sequences (5′-> 3′)	PCR or Sequencing	Origin
1	sGluN-F1	ATC ACC GGC ATC AAC GAC CC	PCR and Sequencing	This study
	sGluN-R1	GCT GCA AAA GCC AGC TGC AT	PCR and Sequencing	This study
2	sGluN-F2	ACT GCT TCA AGT CGG CAT TT	PCR and Sequencing	This study
	sGluN-R2	GGG TCG TTG ATG CCG GTG AT	PCR and Sequencing	This study
3	sGluN-F3	GAG CAG GTG TTC AAG GAT GC	PCR and Sequencing	This study
	sGluN-R3	AAA TGC CGA CTT GAA GCA GT	PCR and Sequencing	This study
4	Uni-primer	TCACAGAAGTATGCCAAGCGA	PCR and Sequencing	*DNA Walking SpeedUp™ Premix Kit
	Chum_NMDAR_5o	TGATGTGGGCCGACTCGTTCC	PCR and Sequencing	This study
	Chum_NMDAR_5i	AGCTCTTTGGCCTCTAGAAGCAGC	Sequencing	This study
5	Uni-primer	TCACAGAAGTATGCCAAGCGA	PCR and Sequencing	*DNA Walking SpeedUp™ Premix Kit
	Chum_NMDAR_3o	CTGGCTGCCTTCCTGGTGC	PCR and Sequencing	This study
	Chum_NMDAR_3i	TTAGCACCATGTACCGCCACATGG	Sequencing	This study

*indicate of the primer in DNA Walking Speedup™ Premix Kit.

### Cloning of NR1 subunit

The DNA fragments were ligated with pGEM®-T Easy Vector using T4 DNA ligase in the pGEM®-T Easy Vector System I kit (Promega, Mannheim, Germany) and then transformed into competent cells (competent cell RBC, HIT-DH5 alpha High108). The transformations were plated, grown overnight at 37°C, and single-colony miniprepped using an *AccuPrep*® Nano-Plus Plasmid Mini Extraction kit (Bioneer Co., Seoul, Korea). Positive clones were sequenced using T7 and SP6 primer sets and Big Dye Sequencing Kits (Applied Biosystems) on an ABI 3710 automated DNA Sequencer according to the manufacturer’s recommendations (Applied Biosystems, Foster City, CA, USA) at Solgent Co., Korea.

### Expression of NR1 subunit in chum salmon

The real-time PCR was carried out with an Applied Biosystems 7500 Real-Time PCR System (Applied Biosystems, Foster City, CA, USA). The nucleotide sequences of primers and probes are shown in Table 1. The PCR reaction mixture contained 1SYBR premix EX Taq (TaKaRa Biomedicals, Shiga, Japan), ROX Reference Dey II (SYBR premix Extaq, TAKARA, Shiga, Japan), 100 nM each forward and reverse primers and 130 nM of fluorogenic probe. In the assay, several doses of standard cDNA were applied in triplicate, and each sample cDNA prepared from total RNA was applied in duplicate.

### Statistical analysis

The resulting plasmid DNA was alkaline-denatured, and both strands were sequenced using a Dye Terminator Cycle Sequencing Kit and ABI Prism Model 3100 Auto Sequencer (Applied Biosystems, Foster City, CA). The complete sequence of the salmon NR1 subunit was deposited in GenBank (accession number JQ924060). The alignment of multiple protein sequences was performed using the Clustral X and MEGA version 5.0 program (Larkin et al. 2007; Tamura et al. 2007,2011), and homology values (percent of amino acid sequence identity) were calculated by pair-wise alignment. The phylogenic analysis was constructed with the program MEGA 5.0 (Tamura et al. 2007,2011). The stability of internal nodes was assessed by bootstrap analysis (1000 replicates were used for Neighbor-joining).

## Results and discussion

**Figure 1:**
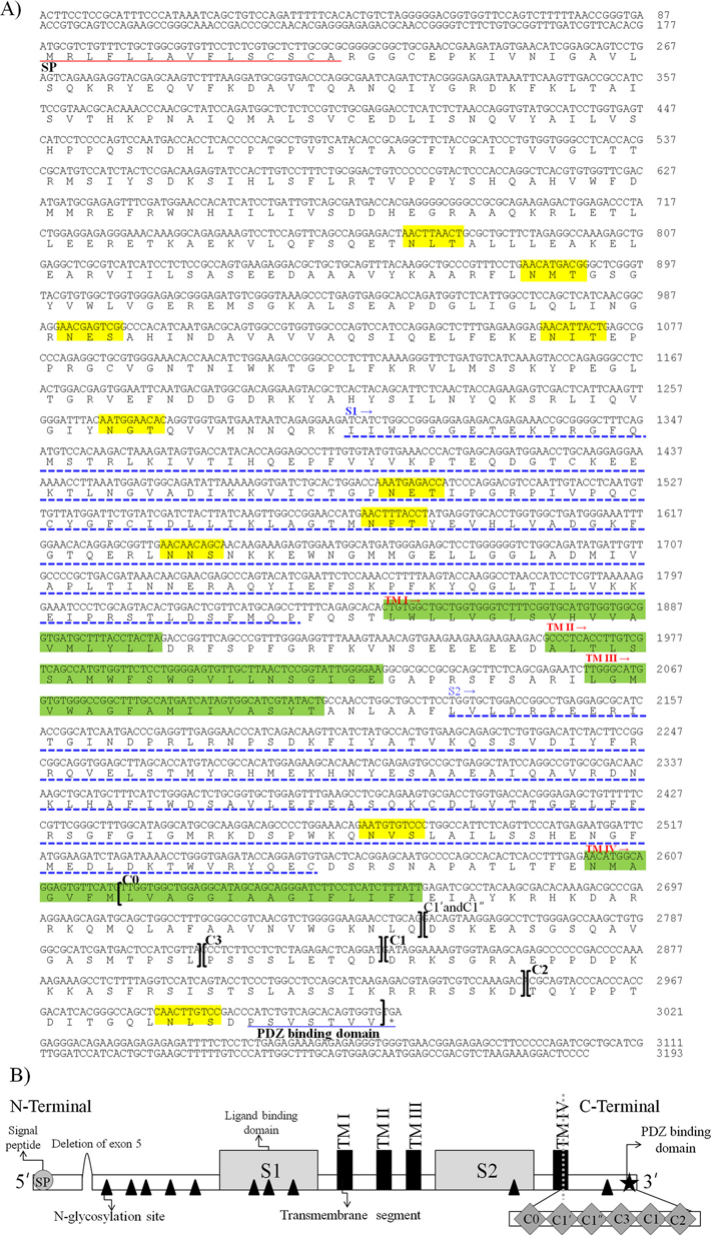
**The nucleotide and amino-acid sequence of NR1 subunit. (A)** and a diagram representing the structure **(B)** of chum salmon NR1 subunit.

**Figure 2:**
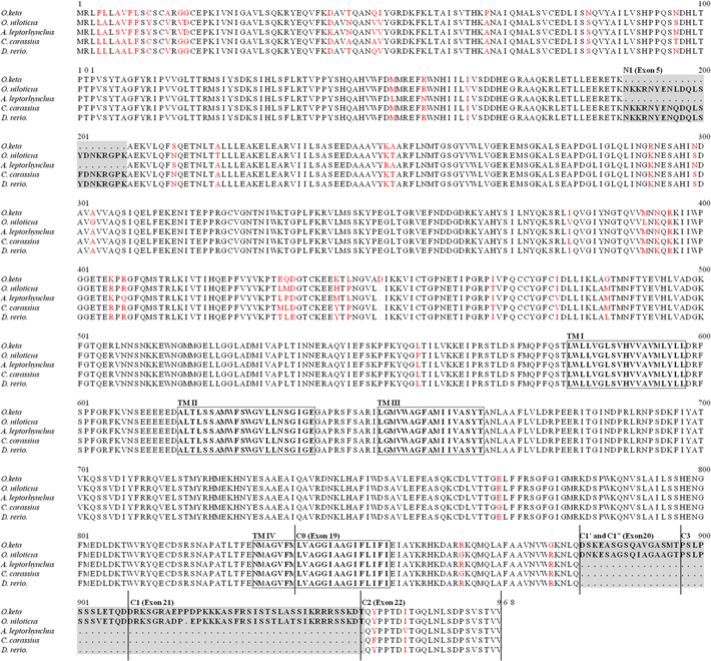
**Molecular characterizations and amino acid sequence comparison of teleost NR1 subunit.** The red character indicated variation sites.

A PCR cloning approach was used to identify a potential chum salmon NR1 subunit expressed in homing chum salmon. Using PCR primers designed from highly conserved NR1 subunit regions of *O. niloticus*, *A. leptorhynchus*, *C. carassius*, and *D. rerio*, the cDNA encoding the NR1 subunit was identified. We were able to isolate distinct cDNA fragments, suggesting that one gene for the NR1 subunit is expressed in the chum salmon. The nucleotide sequence of the cloned cDNA showed a large open-reading frame of 2844 bp in the total length of 3193 bp, and this cDNA contained a coding region encoding 948 amino acids and a stop codon (Figure 1A). The 5′ untranslated region (UTR) and 3′ UTR were region of 1–177 bp and region of 3022–3193 bp, respectively. This cDNA molecule encoded a protein of 948 amino acids displaying up to 90% identity with *O. niloticus*, *A. leptorhynchus*, *C. carassius*, and *D. rerio* (Figure 2).

The structure of the NR1 subunit was first deduced from chum salmon in this study and is shown in Figure 1. The subunit contains one hydrophobic region at the amino terminus (supposedly a signal peptide), four hydrophobic transmembrane regions (TM-I, TM-II, TM-III, and TM-IV), two ligand-binding domains (S1 and S2), and a potential PDZ domain-binding motif at its C-terminus (Cox et al. 2005; Tzeng et al. 2007). Among these regions, TMII is thought to make a hairpin turn within the membrane and line the ion channel, and S1 and S2 are predicted to form the binding domains for glutamate and glycine (Zukin and Bennett 1995). In addition, the S1-S2 binding pockets in NMDA receptors via an evolutionary analysis showed that disruption of domain closure could affect channel activation and may affect the normal behavior of animals (Blaise et al. 2004; Koo and Hampson 2010). Most of the important amino acid residues for ligand binding are conserved in NR1 (Figure 1). The gene structures of mammalian, tilapia, and zebrafish NR1 genes are similar; however, they differ in terms of the exon coding for the C-terminal domain by spliced variants. For example, the teleost NR1 genes lack the C2 sequence (Bottai et al. 1997,1998; Harvey-Girard and Dunn 2003; Cox et al., 2005; Kinoshita et al. 2005).

The N-terminal region was more divergent compared to the proteins of four other fish species, but it was still more than 60% identical (Figure 2). However, the region from amino acid 486 to the C-terminal showed the highest degree of identity among the four different proteins; this region contained the putative transmembrane regions as well as the MG^++^-binding domain (Figure 1). The transmembrane topology of the NR1 subunit has four hydrophobic segments (TM-I, TM-II, TM-III, and TM-IV) in the middle of the molecules (Figure 1). These structural characteristics seem to be common for neurotransmitter-gated ion channels, such as the nicotinic acetylcholine receptor (nAChR) channel (Noda et al. 1983), the gamma-aminobutyric acid receptor (GABAR) channel (Schofield et al. 1987), and the glycine receptor (GlyR) channel (Grenningloh et al. 1987). However, the initial four transmembrane segment models for the NR1 subunit may not be correct (Moriyoshi et al., 1991).

Elucidation of the entire structure of the chum salmon NR1 subunit allowed us to confirm the splicing pattern for the generation of NR1 variants in this species. Figure 1 shows a schematic representation of the predicted splicing pattern at the N- and C-terminal regions of the chum salmon NR1 subunit, deduced from information on the structure of the gene and previous experimental data of NR1 splice variants in the zebrafish and tilapia (Cox et al. 2005; Tzeng et al. 2007). The chum salmon shows a splice variant by deletion of N1 (exon 5) in the N-terminal region, but the C-terminal region did not show deletion of any exons (Figure 1). However, several reports have already suggested that the NR1 subunit region has some splice variants in mammals and teleosts resulting from insertion and deletion at the N- and C-terminal regions (Moriyoshi et al. 1991; Sugihara et al. 1992; Nakanishi et al. 1992; Lee-Rivera et al. 2003; Tzeng et al. 2007). Generally, the NR1 subunit is now known to have a total of 22 exons (Hollmann et al. 1993), 3 of which (exons 5, 21, and 22) undergo alternative splicing to generate theoretically create eight NR1 splice variants, and these regions are called N1 (exon 5), C1 (exon 21), and C2 (exon 22) (Zukin and Bennett 1995). The C-terminal region of NR1 shows high homology of C0 (exon 19) and C2 (exon 22) among fish, including chum salmon (Figure 1). In contrast, the region C1′–C′′–C3 (exon 20) and C1 (exon 21) revealed high variability by the inclusion of additional alternatively spliced exons in the chum salmon/*O. niloticus* and in *A. leptorhynchus*/*C. carassius*/*D. rerio*. These data support high variability in the C1′–C1′′–C3-C2 parts in the C-terminal region among species (Figure 1). Alternative usage of exons is frequently tissue- and development-specific, and cassette exons are the most common form of alternatively spliced exons. The general expression patterns of NR1 splice variants in various brain regions have been shown to be conserved between rat and knifefish (Nakanishi et al. 1992; Bottai et al. 1998; Stamm et al. 2000). Therefore, further analysis of various brain regions is required for proof of spliced variant types in the chum salmon.

**Figure 3:**
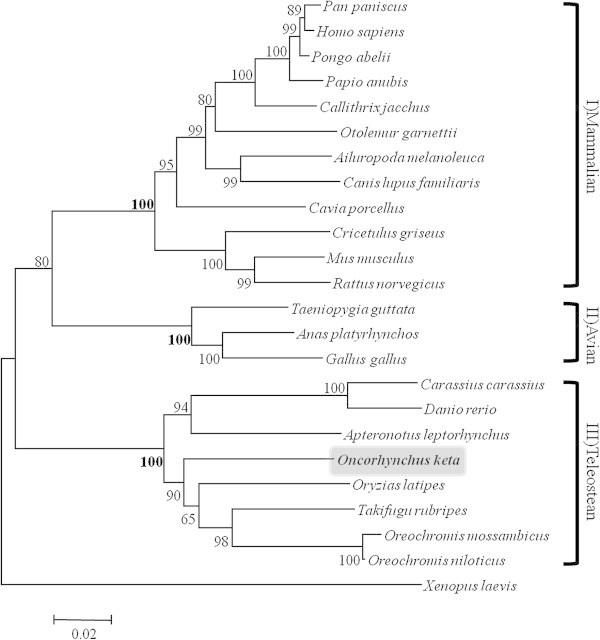
**Neighbor-joining tree based on the NR1 subunit sequences.** The numbers at the nodes are bootstrap values computed using 1000 replications and Kimura’s 2-parameter distance model.

For observed genetic variation of NR1 subunit, we performed phylogenic analysis base on comparative among 12 mammalian, 8 teleostean, and 3 avian from GenBank data. The neighbor-joining tree formed three distinctive clade (Figure 3), mammalian clade (I), avian clade (II), and teleosten clads (III). The mean genetic distances were 1.6% between clade I and clade II, 2.0% between clade II and clade III, and 1.9% clade I and clade III. The NR1 subunit could be reliably classified by phylogenetic analysis with strong bootstrap values (100% of replicates among clade).

**Figure 4:**
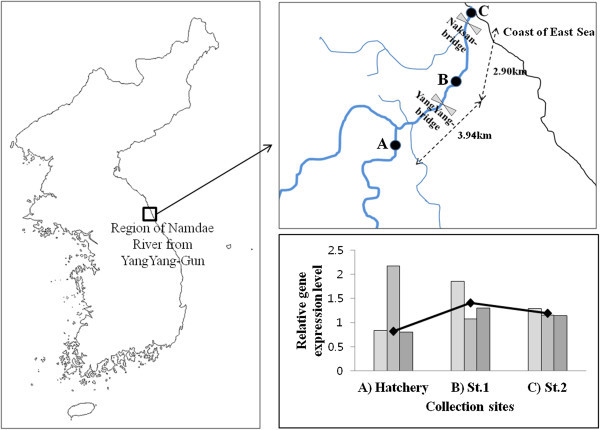
**RT-PCR expression analysis of NR1 in the three collection sites from Korea. A)** Displays the location of study area in the Korea. **B)** Displays the detail information of study site. **C)** Displays the relative gene expression level in the each study site.

The expression pattern of the NR1 was analyzed by RT-PCR (Figure 4). When it has to release chum salmon from hatchery (site A) in the Namdae River, the site B near to the hatchery showed the highest expression of NR1 in brain tissue, but lower expression levels were detected in cite C near to the coast. This is interesting because this is the first evidence of the upregulation of NR1 gene expression in brain of chum salmon during their migration to the sea. If the theory of NMDARs are related to learning and memory (Cull-Candy et al. 2001; Dingledine et al. 1999; Hua and Smith 2004; Waxman and Lynch 2005), our result might be suggests that the NR1 in the brain was related to remember about some contents of natal rivers. However, although reaction of variable expression, we can not conclude that NR1 is to activity about amino acid of the natal river. Therefore, to support of data in this study, we have studies more expression biological analysis on NR1 is to enhance our understanding of important olfactory functions in salmonid species.
